# Comparison of Operative Time and Blood Loss With the FFX® Device Versus Pedicle Screw Fixation During Surgery for Lumbar Spinal Stenosis: A Retrospective Cohort Study

**DOI:** 10.7759/cureus.22931

**Published:** 2022-03-07

**Authors:** Robin Srour

**Affiliations:** 1 Neurosurgery, Hôpitaux Civils de Colmar, Colmar, FRA

**Keywords:** ffx, spine, pedicle screws, blood loss, operative time, lumbar fusion

## Abstract

Background

Pedicle screw (PS) placement can be associated with soft tissue damage and blood loss. The study objective was to evaluate differences in operative time and blood loss between PS fixation and an implantable facet fusion device in patients undergoing lumbar fusion surgery.

Materials and methods

A retrospective analysis was performed on patients undergoing lumbar fusion surgery with PS fixation or the lumbar Facet FiXation (FFX) device. Procedures were performed by the same surgeon at a single institution. The PS group included patients from 2016 and the FFX group included patients from 2018. Variables including age, sex, levels operated on, operative time, and operative blood loss were collected.

Results

A total of 70 patients were included in the study. Twenty-eight in the PS arm and 42 in the FFX arm. The PS group had a mean age of 67.5 ± 9.3 years compared to 70.4 ± 11.5 years for the FFX group. The PS group had a higher percentage of females (57.1%) versus the FFX group (31.0%); p = 0.025. Mean number of levels operated on were similar between the PS and FFX groups (2.3 ± 1 .1 vs. 2.2 ± 1.0, respectively; p = 0.89). Mean operative time was significantly longer for the PS group versus the FFX group (152.5 ± 39.4 vs. 99.4 ± 44.0 minutes; p < 0.001). Mean operative blood loss was significantly greater for the PS group versus the FFX group (446.5 ± 272.0 vs. 251.0 ± 315.9 mL; p < 0.01). Differences were independent of the number of levels operated on.

Conclusion

Placement of the FFX device is associated with a significant reduction in operative time and blood loss compared to PS fixation in patients undergoing spinal fusion surgery.

## Introduction

Pedicle screw (PS) fixation following decompression is currently considered the standard technique for achieving fusion and spinal stability in patients with lumbar spinal stenosis (LSS) [1]. Placement of PSs via open lumbar surgery using a posterior approach is associated with significant soft tissue damage and blood loss [2,3]. Procedural associated blood loss with PS placement increases the risk of postoperative infections, hematoma formation within the spinal canal, and blood transfusion [4,5]. The use of PS constructs can also result in adjacent level degeneration due to the rigidity produced by this approach and the resultant overload of anatomical structures [6].

The lumbar Facet FiXation (FFX®) device (SC Medica, Strasbourg, France) is a new implantable facet spacer intended to increase foraminal space and promote fusion while reducing load projections on adjacent lumbar levels [7]. The device is designed to prevent facet motion and post-laminectomy instability in patients with LSS while avoiding the rigidity associated with conventional PS constructs. The FFX spacer is a titanium-constructed, D-shaped device with a serrated surface which facilitates device stabilization. The device is surgically positioned between the facet joints, with its apex oriented anteriorly. Bone graft material is placed inside and posterior to the device in order to facilitate fusion.

As a result of the ability to place the FFX device without the use of imaging, it theoretically reduces operative time compared to PS since the latter requires imaging to ensure proper placement. The above combined with the reduced surgical exposure requirement for the procedure to place the FFX device versus PS potentially translates to a reduction in operative blood loss. In order to assess the above, the present retrospective cohort study was conducted with the aim of comparing differences in operative time and blood loss between procedures performed with the FFX device versus PS in patients with LSS.

A preprint of this article was previously posted to the medRxiv server on May 13, 2020 (https://www.medrxiv.org/content/10.1101/2020.05.09.20096651v1).

## Materials and methods

**Figure 1:**
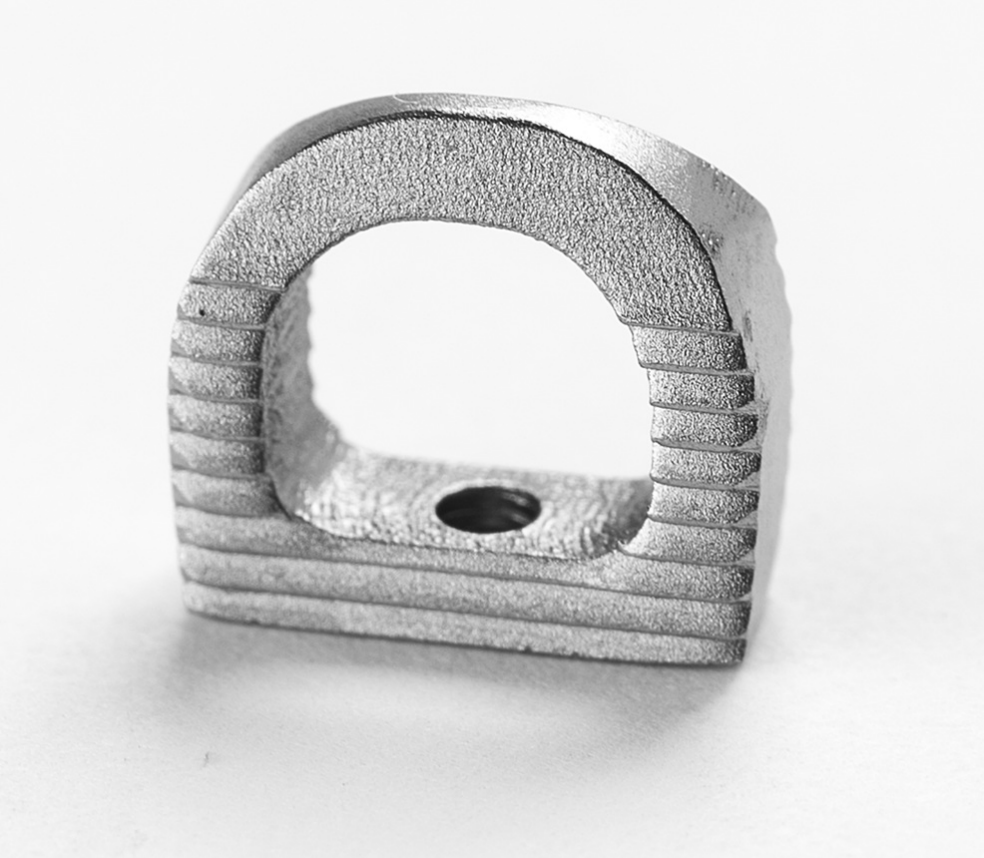
Lumbar Facet FiXation (FFX) device.

The present study was a non-randomized, retrospective cohort analysis of patients with LSS undergoing spinal fusion surgery via PS fixation or the FFX device during two separate time periods. The study was approved by the Institutional Review Board at Hôpitaux Civils de Colmar (approval number 17/002). The PS group consisted of all the patients with LSS operated on in 2016 who underwent an open technique for decompression, specifically laminectomies, concomitant to PS fixation. The FFX group included all the patients with LSS operated on in 2018 who underwent open laminectomies concomitant with FFX fixation. These two time periods were selected as a result of PS being used as the primary implant for LSS-associated surgeries during 2016 and the transition to only using the recently introduced FFX device was completed prior to 2018. In order to avoid the potential for operator and institutional bias, the study only included procedures performed by a single surgeon at the same institution (Figure 1). 

Exclusion criteria included radiographically confirmed damage of the vertebral body in the segment of concern in the lumbar spine, spinal fracture, spondylodiscitis, spinal tumor, osteoporosis, Paget disease, osteomalacia or other metabolic bone disorders, more than five-level involvement, morbid obesity, pregnancy, or known allergy to titanium or titanium alloys. Additional exclusion criteria included patients having both PS and FFX devices implanted during the same operative procedures, patients with previously fused facet joints, patients requiring removal of previously placed hardware, and patients having complications during surgery.

Data collected included patient sex and age, number of spinal levels operated on, operative time, and blood loss. The objective of the present study was to identify potential differences in mean total operative time and procedural blood loss for the PS and FFX patient populations overall and by number of levels operated on. An analysis of potential differences in clinical or radiological outcomes between the two patient groups was outside of the scope of the present study. 

Operative techniques

**Figure 2:**
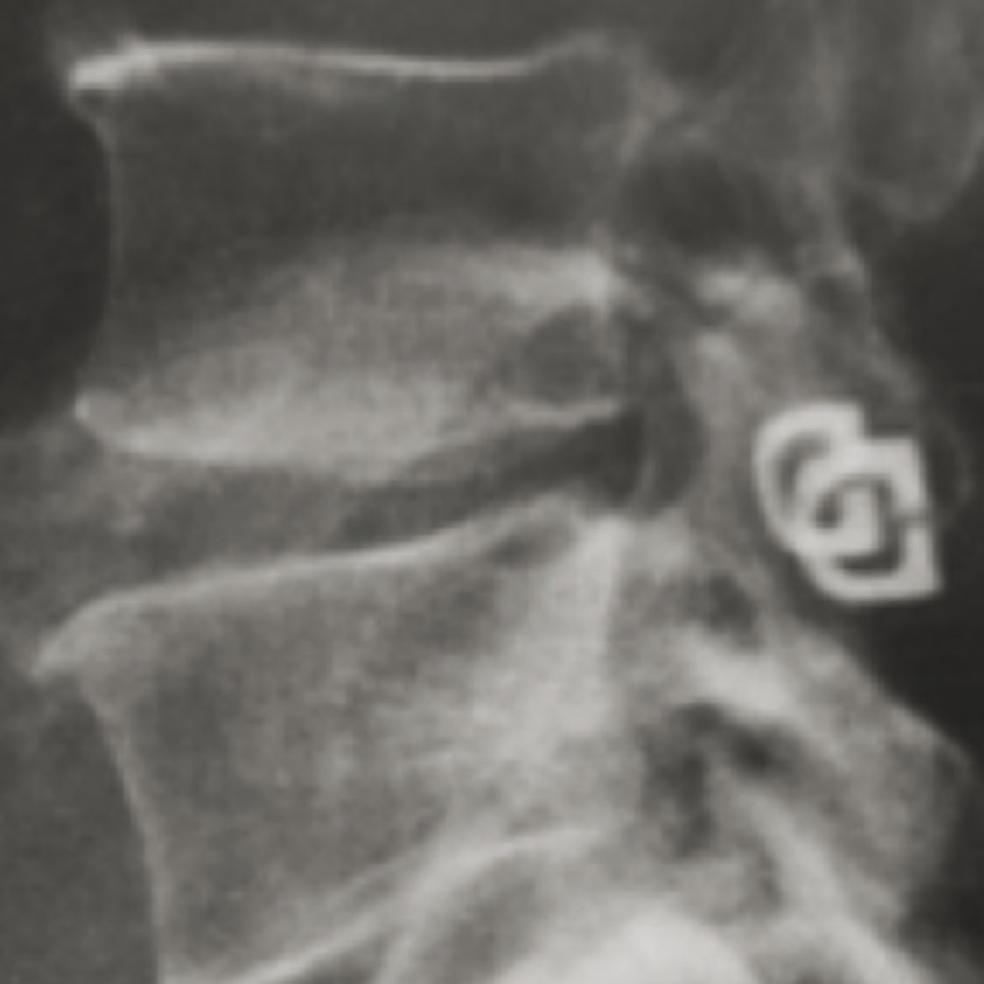
FFX devices inserted into the facet joints on the right and left sides of the spine. FFX, Facet FiXation.

For FFX procedures, open laminectomies were performed, followed by FFX fixation. The laminectomy procedure included complete ablation of the laminas facing the canal narrowness followed by an internal facetectomy with radicular recalibration. Tracking of the facet joints line spacing was performed with a facet chisel followed by a reviving of the facet joints with a rasp to promote fusion. Two implants were used per level. After connecting the FFX implant onto the facet holder, bone graft material was inserted into the empty space of the device. While attached to the facet-holders and at the entry of the facet joint lines, the devices were inserted into the facet joint simultaneously on the right and left sides, under direct visualization (Figure 2). The devices were then pushed into place using a supplied impactor and positioned appropriately. The above was followed by a laminectomy and bone graft material added posterior to the inserted implants. For PS procedures, open laminectomies were performed in a similar manner as was done FFX fixation with the exception of preserving the facets. PSs placement was performed using fluoroscopic guidance. Surgical wounds were closed and sutured per standard routine following completion of the procedures.

Statistical analysis

Statistical differences in sex and the proportion of the number of levels between FFX and PS for any of the levels were assessed using Fisher's exact test for counts data (two-sided). Statistical differences in the mean number of levels operated on between the two groups were assessed using the two-sample t-test (two-sided). Statistical significance for differences in surgery duration and blood loss between FFX implants and PS fixation procedures were assessed using the unpaired Wilcoxon two-sample test with a linear regression model used to adjust differences in the number of lumbar interbody cages placed between the two groups. All analyses were performed using R v3.6.2. 

## Results

A total of 70 patients met the study criteria during the two separate time periods identified. This included 28 patients who had PS fixation in association with surgery for LSS during 2016 and 42 patients who underwent the FFX procedure for the same indication in 2018. A total of 31 patients were excluded consisting of 15 patients that had both PS and FFX devices placed during the same procedure, five FFX patients who only had a single unilateral device placed since one facet was previously fused, three FFX and four PS patients who required removal of material from a previous surgical procedure, and four PS patients who experienced complications during the surgery (i.e. dural tears). None of the patients receiving the FFX device experienced intraoperative complications. 

Patient demographics for the two study groups were similar for all parameters except for sex, with the PS group having a greater percentage of females (57.1%) compared to the FFX group (31.0%), p = 0.025. Mean age for patients undergoing PS fixation was 67.5 ± 9.3 years (range 42.7-87.5) compared to 70.4 ± 11.5 years (range 49.7-86.6); p = 0.18). The mean number of levels operated on were 2.3 ± 1.1 for the PS group versus 2.2 ± 1.0 for the FFX group; p = 0.89 (Table 1). The number of levels operated on ranged from one to four for both groups with each group having implants placed at various levels from L2/L3 through L5/S1; p = 0.49. Implants were placed at L4/L5 for 24 of 28 patients (85.7%) in the PS group and 36 of 42 patients (85.7%) in the FFX group. The mean number of implants per patient was greater in the PS group (6.5 ± 2.2, range 4-10) compared to the FFX group (4.38 ± 2.1, range 2-8) because of the need to place four screws for the initial level operated on in the PS group. Five patients (11.9%) in the FFX and 17 patients (60.7%) in the PS group had lumbar interbody cages placed concomitantly during the procedure. 

**Table 1:** Comparison of the number of spinal levels operated on There was no significant difference (p = 1.0) between the two groups for the number of surgical levels using a two-sample Z-test for proportions in each level, with multiple testing adjustment using the Bonferroni method. FFX, Facet FiXation.

Number of surgical levels	Pedicle screws (n = 28)	FFX devices (n = 42)
One level	9 (32.1%)	13 (31.0%)
Two levels	9 (32.1%)	12 (28.6%)
Three levels	4 (14.3%)	12 (28.6%)
Four levels	6 (21.4%)	5 (11.9%)

**Figure 3:**
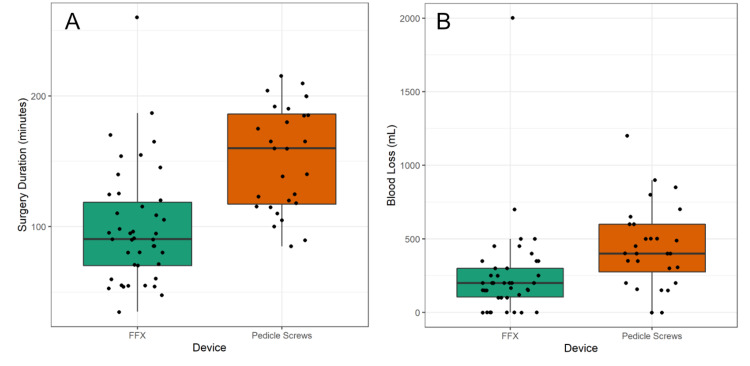
Box plots comparing (A) operative time and (B) blood loss for patients receiving FFX device vs. PS. The difference between the FFX device vs. PS was significant for both operative time (p < 0.001) and for blood loss (p < 0.01). FFX, Facet FiXation; PS, pedicle screw.

Mean operative time was significantly longer by 53.1 minutes for the PS group versus the FFX group (152.5 ± 39.4 vs. 99.4 ± 44.0 minutes; p < 0.001) (Figure 3). The difference between FFX and PS relative to operative time remained significant even when adjusted for the difference in placement of interbody cages between the two groups (p < 0.001). The significant difference in less operative time associated with the FFX device was independent of the number of levels operated on (Table 2).

**Table 2:** Mean operative time for PS vs. FFX device placement procedures Operative time reported in minutes ± SD. SD, standard deviation; PS, pedicle screws; FFX, Facet FiXation.

Number of surgical levels	PSs (n = 28)	FFX devices (n = 42)	p-Value (FFX vs. PS)
All levels	152.5 ± 39.4	99.4 ± 44.0	p < 0.001
One level	124.4 ± 35.3	90.6 ± 38.4	p = 0.03
Two levels	133.8 ± 17.3	133.8 ± 38.9	p = 0.005
Three levels	197.0 ± 15.4	121.5 ± 50.5	p = 0.02
Four levels	191.0 ± 8.6	104.8 ± 27.5	p = 0.008

Mean operative blood loss was also significantly greater for the PS group compared to the FFX group. Patients undergoing PS fixation experienced an average 195.5 mL greater blood loss per procedure than those in the FFX group (446.5 ± 272.0 mL for PS vs. 251.0 ± 315.9 mL for FFX; p < 0.01) (Figure 3). The difference between FFX and PS relative to blood loss remained significant even when adjusted for the difference in placement of interbody cages between the two groups (p < 0.01). Mean blood loss was significantly less with the FFX device for all numbers of levels operated on with the exception of single-level procedures where there was only a trend for less blood loss compared to PS fixation (Table 3).

**Table 3:** Mean operative blood loss for PS vs. FFX device placement procedures Blood loss reported in mL ± SD. SD, standard deviation; PS, pedicle screw; FFX, Facet FiXation.

Number of surgical levels	PSs (n = 28)	FFX devices (n = 42)	p-Value (FFX vs. PS)
All levels	446.5 ± 272.0	251.0 ± 315.9	p < 0.01
One level	350.0 ± 257.1	247.7 ± 201.3	p = 0.30
Two levels	350.9 ± 183.1	170.8 ± 145.0	p = 0.05
Three levels	720.0 ± 255.0	367.9 ± 497.5	p = 0.02
Four levels	499.2 ± 196.8	171.2 ± 183.2	p = 0.05

## Discussion

The present study demonstrated that the use of the FFX device in conjunction with posterior lumbar decompression for patients with LSS results in a significant reduction in both operative time and blood loss compared to PS fixation. These findings were independent of the number of levels operated on or if interbody cages were placed.

Reducing operative time for spinal surgery procedure has several benefits. Radiation exposure is an important consideration related to the use of PS fixation for lumbar fusion since these procedures require intraoperative imaging for guidance. Cumulative exposure to ionizing radiation associated with PS placement has potential detrimental long-term effects on surgeons [8,9], with a spine surgeon’s hands and torso receiving the highest radiation doses [10]. Minimally invasive procedures to place PSs have also been shown to require greater use of fluoroscopy compared to open procedure, increasing the potential for radiation-induced complications [11]. The decreased operative time and absence of the need for fluoroscopy associated with the placement of the FFX device compared to PS fixation would, therefore, be advantageous relative to reducing radiation exposure. Reduced operative time is also associated with decreased procedure and operating-room-related costs as well as enhancing operating room efficiency [12].

Increased blood loss during lumbar spine surgery is associated with the increased need for transfusions, risk of postoperative complications, and length of stay following spinal surgery. Postoperative anemia has been shown to be associated with prolonged hospital stay and costs following lumbar spine procedures [13]. Additionally, receiving allogenic blood transfusions increases the risk of developing postoperative infections following spine surgery and prolonged length of stay [14]. Another concern with regard to perioperative bleeding in spinal surgery is the risk of spinal epidural hematoma formation, which might lead to spinal cord or cauda equina compression [5]. The observed reduction in blood loss with the FFX device versus PS fixation in the present study could potentially lead to a decreased need for transfusions and associated sequalae. Additional clinical studies are needed to confirm this.

A recent prospective, single-arm, multicenter study assessed clinical outcomes with the FFX device related to pain reduction and fusion rate in 53 patients with LSS or facet syndrome with and without concomitant posterior lumbar interbody fusion procedures [7]. Mean visual analog scale leg and back pain scores significantly improved from 5.57 to 2.09 (p < 0.001) and 5.74 to 3.13 (p < 0.001), respectively, between the preoperative and one-year follow-up assessments. Mean Oswestry Disability Index (ODI) scores also significantly improved from 44.7% to 24.0% (p < 0.001) during the same time period. Facet fusion occurred with 86.3% of device placements after 12 months. There was one (0.5%) asymptomatic device migration, and eight devices (3.9%) were considered misplaced.

In addition to the reduced invasiveness and reduced operative time associated with the placement of the FFX device compared to PS fixation, the ability of the FFX device to provide less rigid fixation and potentially reduce projected loads compared to PS constructs could result in less adjacent segment degeneration and a decreased need for subsequent surgical procedures. While finite element modeling comparing the biomechanical performance of the FFX device to PS fixation both before and after fusion suggests this [15], clinical studies that document reduced reoperation rates for adjacent segment degeneration in association with the use of the FFX device are needed.

There are several potential limitations associated with the present study. The use of a non-randomized, retrospective study design over two separate time periods hinders the ability to make definitive conclusions when comparing these two instrumented lumbar spine procedures since confounding factors, including those that could have influenced operative time and blood loss, may have impacted the outcomes observed. No attempt was made in the present study to match or stratify the two patient populations for these or other demographic factors. Additionally, the lack of detailed information regarding patient characteristics, medical history, and the criteria for the decision to operate creates the potential for selection bias. Finally, the limitation of the patient population to procedures performed by a single individual to avoid potential surgeon-to-surgeon differences in operating time limits the ability to generalize the results. Expanding the present analysis to include additional surgeons and institutional settings would provide further evidence supporting the findings of the current study. 

## Conclusions

Lumbar PS placement using an open surgical approach can result in intraoperative blood loss with an associated increased risk of postoperative infections, hematoma formation, and blood transfusion. The present study demonstrated a statistically significant reduction in both operative time and procedural blood loss associated with the FFX device compared to PS fixation in patients undergoing posterior lumbar decompression with fusion. Additional studies are needed to better understand differences in biomechanical performance between the FFX device and PS fixation and the potential for development of adjacent segment degeneration following lumbar decompression surgery.
